# Control Work

**Published:** 1897-11

**Authors:** 


### CONTROL WORK.

---

*Belgium.* The Director-General of Agriculture has issued an order that all bovines over three months old must be marked with a tag on the ear, in order that they may better control tuberculosis among these animals.

*South Africa.* The Government of Cape Colony has issued directions for the inoculation against rinderpest either with pure bile, Dr. Koch's method, or with glycerinated bile, according to Dr. Edington's method. We clip the following from our esteemed contemporary, the *Agricultural Journal,* of Cape Colony :

PROF. KOCH'S METHOD : Experience has shown that certain galls, although physically correct in character and appearance, do communicate rinderpest to a certain number of cattle when they are inoculated with it; it follows, therefore, that the gall which will thus communicate the disease by inoculation would also be capable of communicating infection in the ordinary way. Hence the necessity of exercising extreme care when inoculating a clean herd of cattle, and the following directions should be attended to carefully :

1. In selecting bile, none but that which shows a deep green color with a white froth and free from sediment should be accepted, and, where practicable, several different galls should be mixed together. This modifies and equalizes the strength and safety of the gall. The selected green gall should be kept separately for at least twelve hours. If any gall smells or changes color after keeping, it must be rejected. The good galls are then mixed together before being sent out.

2. Gall is considered more effective, and gives the best results when obtained from a sick beast on the tenth day after artificial inoculation, or the fourth or fifth day after the visible symptoms have become apparent in natural infections. Earlier or later gall should be avoided, and gall taken from an animal which has died, unless the duration of the disease is known, is not to be recommended.

3. The gall should be used on the second day after being drawn, if practicable, but, if kept cool, either by placing the bottles in icechests or wrapping them in wet cloths and placing them in a cool draught, it may be kept fresh and safe up to the third or even the fourth day.

4. Both before and after the gall is placed in bottles they should be disinfected, and also the cloths that are wrapped around them before being sent to the inoculator.

5. The inoculator should not extract the gall from the affected animal himself, nor come near to the infected cattle; but, if necessary for him to do so, he should change his clothes and boots and disinfect his hands well before commencing to inoculate; also see that everyone handling the cattle has been thoroughly disinfected.

6. As it is never certain when inoculating within an infected district that the herd is completely free from infection, the needle of the syringe should be cleaned and disinfected in a 5 per cent. solution of carbolic acid or alcohol after each inoculation, and the skin of the animal at the seat of the inoculation should be cleaned and disinfected, both before and after the operation with Jeye's fluid or other similar disinfecting solution.

7. The small cup or vessel containing the gall, from which the syringe is repeatedly filled, should be kept covered with a small piece of linen or calico, wrung out in the disinfecting solution, and the operator's hands and syringe should be frequently disinfected.

8. As infection may be in any herd unknown to the operator he should disinfect his hands and clothes well after inoculating each herd, and, for the same reason, it is not desirable to have the same boys to assist in catching different herds of cattle.

Duncan Hutcheson,
Colonial Veterinary Surgeon.

Dr. Edington's Method: 1. The bile to be used must be taken from an animal immediately after death at full period of disease, or from one which is killed in the last stage of collapse.

2. Any biles can be used, except those which have become putrid and thin. Light yellow-colored biles should also be discarded.

3. All the biles taken on one day are to be dealt with separately, and mixed with glycerin in the proportion of two parts of bile to one part of glycerin. All the glycerinated biles are thereafter to be mixed together and labelled, showing the date of adding the glycerin.

4. No bile should be used for inoculation until eight days after having been mixed with glycerin.

5. The bile mixture should be kept at ordinary room-temperature and not exposed to freezing.

6. The bile, immediately previous to being used for inoculation, must be shaken up, to insure that any precipitate shall be uniformly distributed through it.

7. The dose for inoculation for cattle of average weight is 20 cubic centimetres, which must be injected into the brisket or dewlap. For very young calves 15 centimetres suffice, but for old and heavy cattle 25 centimetres may be given.

8. The second inoculation is made on the tenth day subsequently, and should consist of 0.1 cubic centimetres of virulent blood mixed or suspended in 5 centimetres of water. A convenient method of measurement consists in adding half of a fluidounce of blood to a quart bottle (26 ounces) of sterile water. Of such a mixture of blood and water 5 cubic centimetres are used for injection into the dewlap.

Note.—The virulent blood should be taken from a characteristic case of the disease, and preferably at the stage when the symptoms are beginning to be evident.

After the second inoculation is completed, one may look for the occurrence of some cases of sickness among the animals between the eighth and tenth day after blood-inoculation, and, if any such occur, they should be isolated from the herd.

If, however, after fourteen days no sickness has shown itself, the farmer may rest satisfied that no more will sicken.

ALEXANDER EDINGTON, M.D.,
Director of the Bacteriological Institute.

*New York.*—There are sixty milk-inspectors throughout this Commonwealth. The chief adulteration is by watering and skimming; 600 cases in 1895, 400 in 1896, and 400 in 1897 up to date is the record of cases in violation of the laws. Texas-fever, rabies, and anthrax have come under the notice of the Commissioner of Agriculture during the year; Texas-fever from cattle with ticks from the quarantine district. The losses this year from these diseases have been reduced to a minimum.

---

Drs. Leonard Pearson, M. E. Conard, James A. Waugh, J. B. Irons, and W. Horace Hoskins are among the list of lecturers before the Farmers' Institute in Pennsylvania during the sessions of 1897 and 1898.

